# 

**Published:** 1878-12

**Authors:** 


DECEMBER, 1878.

TWENTY-FIFTH YEAR—No 300.

### HALL'S

### Journal of Health.

PUBLISHED AT 141 EAST EIGHTH STREET,
(Near Broadway), NEW YORK.

E. H. GIBBS, A.M., M.D., Editor.

AGENTS FOR THE TRADE:
AMERICAN NEWS COMPANY AND NEW YORK NEWS COMPANY

Terms, $1.50 a Year, or $1.00 for Eight Months. Postage paid.

**SINGLE NUMBER, 15 CENTS.**

Couch & Johnston, Printers, 11 Frankfort Street, New York.

